# Prospective Cohort Study with Active Surveillance for Fever in Four Dengue Endemic Countries in Latin America

**DOI:** 10.4269/ajtmh.13-0663

**Published:** 2015-07-08

**Authors:** Gustavo Dayan, Jose L. Arredondo, Gabriel Carrasquilla, Carmen C. Deseda, Reynaldo Dietze, Kleber Luz, Maria Selma N. Costa, Rivaldo V. Cunha, Luis C. Rey, Javier Morales, Humberto Reynales, Maria Miranda, Betzana Zambrano, Enrique Rivas, Pedro Garbes, Fernando Noriega

**Affiliations:** Clinical Research and Development, Sanofi Pasteur, Sao Paulo, Brazil; Instituto Nacional de Pediatria, Unidad de Apoyo a la Investigación Clínica, Mexico City, Mexico; Centro de Estudios de Investigación en Salud, Fundación Santa Fe de Bogotá, Bogotá, Colombia; Caribbean Travel Medicine Clinic, San Juan, Puerto Rico; Nucleo de Doenças Infecciosas, Universidade Federal do Espírito Santo, Vitória, Espírito Santo, Brazil; Hospital Infantil Varela Santiago, Universidade Federal do Rio Grande do Norte, Natal, Rio Grande do Norte, Brazil; Hospital Universitário, Universidade Federal de Goiás, Goiania, Goias, Brazil; Hospital Universitário, Universidade Federal de Mato Grosso do Sul, Campo Grande, Mato Grosso do Sul, Brazil; Instituto de Biomedicina, Unidade de Pesquisas Clínicas, Universidade Federal do Ceará, Fortaleza, Ceará, Brazil; Clinical Research Puerto Rico, Guayama, Puerto Rico; Centro de Atención e Investigación Médica, Bogotá, Colombia; Clinical Research and Development, Sanofi Pasteur, Bogotá, Colombia; Clinical Research and Development, Sanofi Pasteur, Montevideo, Uruguay; Clinical Research and Development, Sanofi Pasteur, Mexico City, Mexico; Clinical Research and Development, Sanofi Pasteur, Swiftwater, Pennsylvania

## Abstract

To prepare for a Phase III dengue vaccine efficacy trial, 20 investigational sites were selected for this observational study to identify dengue infections in a closed cohort (*N* = 3,000 children 9–16 years of age). Of 255 acute febrile episodes experienced by 235 children, 50 (21.3%) were considered serologically probable dengue, and 18 (7.7%) were considered virologically confirmed (i.e., dengue NS1 antigen positive) dengue cases. Considering the disease-free and at-risk period from study start to onset of symptoms, the overall incidence density of acute febrile episodes was 17.7 per 100 person-years of follow-up, ranging from 15.3 in Colombia to 22.0 in Puerto Rico. This study showed that all sites were capable of capturing and following up acute febrile episodes within a specific timeframe among the established cohort and to detect dengue cases.

## Introduction

Dengue is an endemic disease in the Caribbean, Central and South America, and Mexico, with an increasing incidence over the last decades.^1^ The increasing morbidity and mortality of dengue in the Americas in recent decades are well documented, as is its economic impact, and in several countries this increase has been associated with a modification of the age distribution of cases.^1^–^5^ This increased number of cases has been attributed to various factors, including inefficacy of the *Aedes aegypti* eradication program, population growth, increased urbanization, and climatic changes.^6^,^7^ In 2013, nearly 2.3 million cases of dengue disease, including over 37,000 cases of severe dengue and more than 1,200 dengue-related deaths, were reported in Latin America.^8^

This increasing burden illustrates the limited effectiveness of existing disease prevention methods, based on mosquito control and personal protection, and highlights the need for a vaccine as part of integrated programs. Several dengue vaccine candidates are in development.^9^ Sanofi Pasteur's tetravalent dengue vaccine (CYD) contains four recombinant viruses (CYD-1–4), each with genes encoding pre-membrane and envelope proteins of one of the dengue virus (DENV) serotypes, and non-structural proteins of the attenuated yellow fever 17D vaccine virus.^10^,^11^ This vaccine has been shown to be well tolerated and immunogenic.^12^–^19^ Efficacy of this vaccine was initially assessed in a phase IIb study in Thailand,^20^ and is being evaluated in 10 countries in Latin America and South East Asia in two phase III efficacy trials (www.clinicaltrials.gov; NCT01373281 and NCT01374516).

This work describes the results of a prospective surveillance study in sites that went on to participate in the Latin American phase III efficacy trial. The objectives of this study were to identify acute febrile episodes, describe incidence density, develop and field test operational infrastructure for the efficacy trial, and describe dengue seroprevalence.

## Methods

### Study design.

Between June 2010 and October 2011, a prospective surveillance study in four countries was undertaken in 20 study sites. Sites were located in Brazil (Vitoria, Natal, Goiania, Campo Grande, and Fortaleza); Colombia (Yopal, Aguazul, Acacías, Girardot, La Tebaida, Montenegro, Calarcá, and Armenia); Puerto Rico (Guayama and San Juan in Puerto Rico), and Mexico (Veracruz, Valladolid, Ciudad Mante, Temixco, Tizimin) (Table 1). These sites were selected based on a review of local epidemiological information, suggesting high endemicity in the targeted age range, on the size of this age cohort, and on their estimated capacity to participate in an efficacy trial. The study was designed to continue until ∼1 month before the start of the phase III efficacy trial at each site, with an expected duration of ∼12 months but no longer than 18 months. Therefore, the duration of participation of each subject depended on the date of enrollment and the date of onset of the phase III efficacy trial in each site.

A communication plan was developed and implemented for the duration of the study. This communication plan included a community awareness campaign, meetings with key informants, teachers, parents, healthcare workers, and community members to facilitate community involvement and support, and to provide education on dengue fever and opportunities/benefits of participating in studies and cohort recruitment.

Protocol and study documents were approved by the relevant institutional review boards and ethical committees, and the national regulatory agencies. The study was conducted in accordance with good clinical practices and national regulations. Informed assent was obtained from each participant, and informed consent was obtained from their parents or legal representative.

### Surveillance system – case classification.

The surveillance system was designed to detect all acute febrile episodes of at least 2 consecutive days among cohort subjects. All acute febrile episodes identified in the cohort (defined as 2 or more consecutive days of fever of ≥ 38°C) were considered as suspected symptomatic dengue cases.

Participants and parents were instructed to visit the study site or a dedicated healthcare facility in the event of an acute febrile episode and inform the investigator within 24 hours of fever onset, and were contacted weekly by telephone to ensure that this instruction was followed. Weekly telephone calls also served to identify if unreported febrile episodes had occurred since the last contact and to arrange medical visits as appropriate.

In the event of an acute febrile episode, an acute blood specimen was collected and an additional visit was scheduled to collect a convalescent specimen 7–14 days after the initial visit. The non-structural protein 1 (NS1) enzyme-linked immunosorbent assay (ELISA) antigen was performed on acute blood specimens and immunoglobulin M (IgM)/immunoglobulin G (IgG) ELISA were performed on both acute and convalescent specimens.

Suspected dengue cases with a positive IgM ELISA result or a 4-fold IgG increase were classified as probable dengue, referred to below as serologically probable. Cases with a positive NS1 ELISA antigen were classified as virologically confirmed dengue.

### Dengue seroprevalence.

A blood specimen to assess IgG ELISA was obtained at recruitment and at the end of the study to determine dengue seroprevalence.

### Laboratory testing.

#### Dengue NS1 Ag ELISA.

The PlateliaTM Dengue NS1 Ag kit (Bio-Rad, Hercules, CA) was provided to each site. The assay was run according to manufacturer's instructions. A sample ratio was determined for each sample by dividing the average optical density (OD) of the test sample by the average OD of the cutoff control (tested in quadruplicate). Sample ratios of < 0.5, 0.5–< 1.0, and ≥ 1 were indicative of negative, equivocal, and positive results, respectively.

#### Dengue IgM/IgG ELISA.

The EL1500M Dengue IgM ELISA and EL1500G Dengue IgG ELISA kits (Focus Diagnostics, Cypress, CA) were run according to the manufacturer's instructions. The OD of absorbance for test samples at wavelength 450 nm was divided by the cutoff value for the kit to generate an index value. Positive results were indicated by an index value > 1.0, negative results by an index value < 1.0.

### Statistical methods.

We aimed to recruit 150 subjects per site for a total cohort of 3,000, which was not hypothesis-driven. This sample size was based on the estimated proportion of febrile episodes in the region of 24% and addressing operational aspects to descriptively address the objectives of interest.

Assuming a proportion of febrile episodes of 24% and a sample size of 150 subjects, the probability of observing at least 32 fever events was 0.8 (binomial distribution). Analyses were descriptive with no hypothesis testing. For the main parameters, 95% confidence intervals (CIs) of point estimates were calculated using normal approximation for quantitative data and exact binomial distribution (Clopper-Pearson method) for proportions.^21^ For the incidence density rates, 95% CI were calculated using the Rothman–Greenland method.^22^

## Results

A total of 3,000 children 9–16 years of age (mean: 12.4, SD: 2.0), were enrolled (150 per site), of whom 2,954 (98.5%) completed the study. The study duration for all sites combined was 504 days and mean duration of follow-up of the subjects was 177.6 days.

Of 255 acute febrile episodes, experienced by 235 children (7.8% of participants), 50 displayed a 4-fold increase in IgG titer between acute and convalescent samples, or were positive for anti-dengue IgM and were therefore considered as serologically probable dengue cases, and 18 were positive for dengue NS1 antigen and were considered as virologically confirmed dengue cases (Table 2). That is, 50 of 235 (21.3%) children with acute febrile episodes had serologically probable dengue and 18 (7.7%) had virologically confirmed dengue. Considering only the first case for each participant, and the disease-free, at-risk period from study start to onset of symptoms, the overall incidence density of acute febrile episodes was 17.7 per 100 person-years of follow-up. This varied within the range 15.3 in Colombia to 22.0 in Puerto Rico. The incidence density of virologically confirmed dengue was 1.3 per 100 person-years; that of serologically probable dengue was 3.6 per 100 person-year, and that of serologically probable or virologically confirmed dengue was 4.1 per 100 person-years (Table 2).

Of the 50 serologically probable dengue cases, 11 (22%) were virologically confirmed. Of the 18 virologically confirmed cases, 11 (61.1%) were also serologically probable dengue cases (Table 3). Hence, the predictive negative value of the serological diagnosis was 96.2% and the specificity was 82%.

**Figure 1.**
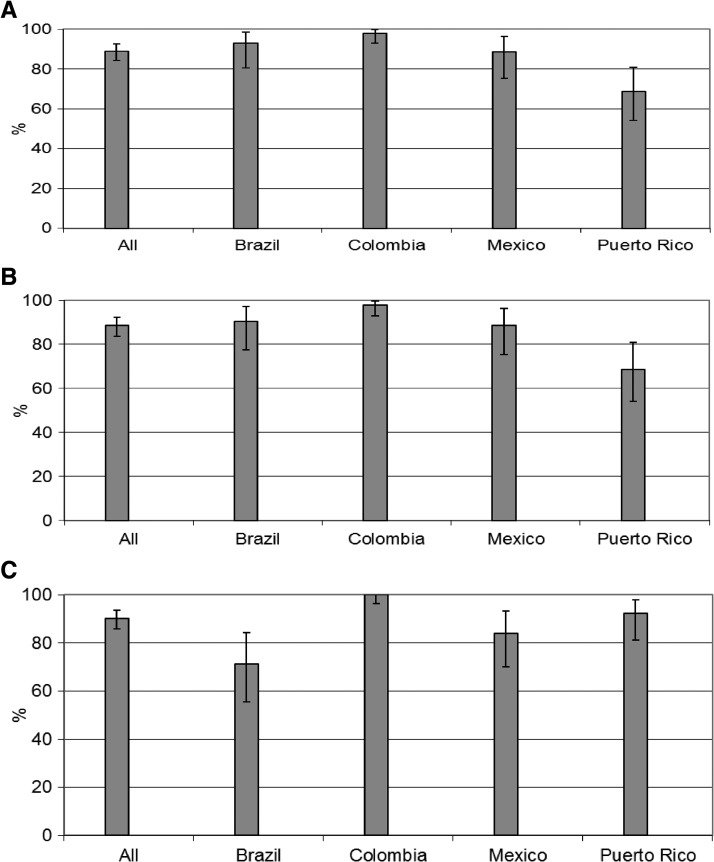
Percentage of subjects with an acute febrile episode who presented for an acute visit (**A**), with acute blood specimens within 5 days of fever onset (**B**), and convalescent specimens within 7–14 days of fever onset (**C**).

Most cases (88.9%) presented for an acute visit within 5 days after fever onset, ranging from 68.6 in Puerto Rico to 98% in Colombia (Figure 1). A similar percentage of subjects had an acute blood specimen drawn within the same interval. Most subjects (90.2%) had a convalescent blood specimen taken 7–14 days after the onset of fever, ranging from 71.4% in Brazil to 100% in Colombia.

The seroprevalence of anti-dengue serum IgG antibodies in the study population was determined at study enrollment and termination. Overall, 70.4% of the study population was positive for dengue at enrollment, ranging from 48.9% in Puerto Rico to 92.5% in Colombia, where almost a half of our study population was enrolled (Table 4). Overall, results at the end of the study were comparable with results at enrollment. Of 44 participants identified as seronegative at baseline and seroconverting during the study, our surveillance system captured 10 (∼23%) who presented with fever. Of these 10, 9 subjects had probable dengue (only serological markers) and 7 subjects had virologically confirmed dengue.

## Discussion

This study was a unique opportunity to train the investigational teams in the field and prepare the local healthcare infrastructure for a phase III dengue vaccine efficacy study in the 9–16 years age group. The teams at all sites showed their ability to actively capture and follow-up acute febrile episodes within the timeframe specified in the protocol to confirm symptomatic dengue cases, which confirmed the feasibility of implementing an active surveillance system to detect and diagnose symptomatic dengue cases in multiple countries in Latin America.

Less than 10% of the detected febrile episodes were virologically confirmed as dengue, illustrating the importance of laboratory confirmation. Serological diagnosis of dengue (IgM/IgG) suggested that 50 cases were probable dengue, but less than one-quarter of these cases were also NS1 antigen positive. This low proportion indicates that many of the positive results from the serological testing could be false positives. Indeed antibodies elicited by other flaviviruses such as yellow fever or Japanese encephalitis, or previous dengue infections can cross-react with the dengue virus leading to false positive reactions.^23^–^26^ Although samples were not tested for other flaviviruses, yellow fever vaccination is included in the national immunization calendars of Brazil and Colombia, and yellow fever is endemic in certain regions in these countries. Nevertheless, serological tests are broadly used and do have value in the diagnosis of dengue. Their strength is their negative predictive value, which gives a high confidence that negative results correctly identifies patients without the disease; i.e., to rule out dengue. In this study the Platelia NS1 ELISA assay was used as a cost-efficient option to virologically diagnose dengue. Although this test is very specific, it has shown to be less sensitive for specimens collected after the first days of illness and for secondary infections.^27^–^29^ In one dengue outbreak in Santos, Brazil, where most cases were secondary infection by dengue 2 virus, NS1 was reactive in 37.7% of RNA+ specimens evidenced by real-time polymerase chain reaction (RT-PCR).^30^ Therefore, virological confirmation should ideally be sought using additional assays to detect the dengue virus, such as RT-PCR. This test, in addition to NS1, should be part of the testing algorithm for virological confirmation in vaccine efficacy trials.

Routine passive surveillance systems do not normally have the capacity to detect all dengue cases.^31^ Therefore, to calculate a more accurate estimate of real incidence, the cases reported through routine surveillance systems need to be multiplied by correction or expansion factors, which represent the degree of underreporting.^32^ Different underreporting rates have been reported in Latin America.^33^–^37^Additionally, higher reporting rates have been documented with the use of an enhanced surveillance system.^38^ The incidence rates observed in our study confirm the high incidence of dengue in children and adolescents from 9 to 16 years of age in these sites.

Our study has several limitations. The timing of participation was not the same for all subjects in the study. Consequently, exposure to dengue may have differed depending on the season of participation in the study. For example, in Colombia in 2010, the dengue incidence peaked in the first half of the year, with an important drop of cases reported in the second half. Study participants from Colombia were included mostly in September 2010 and followed up until May 2011, considered as a year with low incidence of dengue^39^). Differences in incidence should therefore be interpreted with caution, and although our results suggest that incidence may be higher than those reported through routine surveillance systems, accurate comparisons cannot be made because passive surveillance data for the same age group and catchment areas were not collected. This study has shown the capacity of the surveillance system to rapidly detect and obtain acute specimens on the majority of febrile cases, however information on the source of case detection (e.g., self-reported or weekly phone contact) was not systematically collected and could not be analyzed. Another limitation was the lack of testing for other flaviviruses that may have been helpful in the case of samples that were showed a serological response to dengue, but were negative in the virological assay. Finally, it was not meaningful to calculate incidence by site because of the small number of dengue cases by site.

The successful implementation of an active surveillance system and the virologically confirmed dengue activity in all countries in our study confirmed the suitability of these investigational sites to participate in the phase III efficacy study that enrolled more than 20,000 participants from 2011, and is scheduled to conclude in 2014.

## Figures and Tables

**Table 1:** Population, number of laboratory-confirmed cases, and incidence in the provinces/states with study sites during the years the study was conducted

Site	Province/state	2010			2011		
		Population	No. of cases	Incidence rate/100,000	Population	No. of cases	Incidence rate/100,000
Brazil*†							
Vitoria	Espirito Santo	1,847,561	6,621	358.36	1,871,187	11,556	617.58
Natal	Rio Grande do Norte	3,264,647	1,179	36.11	3,302,061	4,453	134.86
Goiania	Goias	6,155,266	24,896	404.47	6,250,462	6,685	106.95
Campo Grande	Mato Grosso do Sul	2,486,257	22,343	898.66	2,520,305	3,081	122.25
Fortaleza	Ceara	8,569,783	13,817	161.23	8,642,630	56,714	656.21
Colombia‡§							
Yopal	Casanare	325,621	2,190	672.56	331,734	401	120.88
Aguazul	Casanare	325,621	2,190	672.56	331,734	401	120.88
Acacias	Meta	870,921	5,600	643.00	888,802	1,610	181.14
Girardot	Cundinamarca	2,477,036	4,251	171.62	2,517,215	465	18.47
La Tebaida	Quindio	549,662	9,713	1,767.09	552,755	224	40.52
Montenegro	Quindio	549,662	9,713	1,767.09	552,755	224	40.52
Calarca	Quindio	549,662	9,713	1,767.09	552,755	224	40.52
Armenia	Quindio	549,662	9,713	1,767.09	552,755	224	40.52
Mexico¶‖							
Temixco	Morelos	1,803,340	1,508	83.62	1,827,187	785	42.96
Veracruz	Veracruz	7,712,247	1,214	15.74	7,791,801	1,886	24.20
El Mante	Tamaulipas	3,334,664	579	17.36	3,376,515	99	2.93
Tizimin	Yucatan	1,980,690	2,525	127.48	2,009,160	6,197	308.44
Valladolid	Yucatan	1,980,690	2,525	127.48	2,009,160	6,197	308.44
Puerto Rico**††							
San Juan	Puerto Rico	3,721,208	9,883	265.59	3,686,580	1,458	39.55
Guayama	Puerto Rico	3,721,208	9,883	265.59	3,686,580	1,458	39.55

* Projeção da população do Brasil por sexo e idade para o período 2000–2060: http://www.ibge.gov.br/home/estatistica/populacao/projecao_da_populacao/2013/default_tab.shtm.

† DATASUS Tecnologiada informacao a servicio do SUS: http://tabnet.datasus.gov.br/cgi/tabcgi.exe?sih/cnv/niuf.def.

‡ DANE/Colombia. Available at: http://www.dane.gov.co/index.php/poblacion-y-demografia/proyecciones-de-poblacion [Update: February 2014].

§ National Institutes of Health/SIVIGILA/Colombia: http://www.ins.gov.co/lineas-de-accion/Subdireccion-Vigilancia/sivigila/Paginas/sivigila.aspx [Update: February 2014].

¶ CONAPO, Projections Census 2010: Mexican Population Projections 2010–2050: http://www.conapo.gob.mx/es/CONAPO/Proyecciones_de_la_Poblacion_2010-2050.

‖ DGE/Secretaría de Salud: http://www.epidemiologia.salud.gob.mx/dgae/infoepid/intd_informacion.html.

** U.S. Census Bureau, Population Division; Annual Estimates of the Population for the United States, Regions, States, and Puerto Rico: April 1, 2010 to July 1, 2013: http;//www.census.gov/popest/.

†† Dengue Surveillance Weekly Report, CDC Dengue Branch and Puerto Rico Department of Health: http://www.salud.gov.pr/dengue/Pages/estadisticasmasrecientes.aspx.

**Table 2:** Number of cases and incidence density* of symptomatic dengue recorded during the trial

Countries	*N*	Subjects with at least one			Incidence density (95% confidence interval) per 100 person-years				
		Acute febrile episode	Serologically probable dengue	Virologically confirmed dengue	Acute febrile episodes	Serologically probable dengue	Virologically confirmed dengue	*Either* Serologically probable *or* virologically confirmed	*Both* Serologically probable *and* virologically confirmed
All	3000	235†	50	18	17.7 (15.6; 20.1)	3.6 (2.7; 4.8)	1.3 (0.8; 2.1)	4.1 (3.2; 5.4)	0.8 (0.4; 1.4)
Brazil	750	42	15	5	20.5 (15.2; 27.8)	7.2 (4.3; 11.9)	2.4 (1.0; 5.7)	7.7 (4.7; 12.5)	1.9 (0.7; 5.1)
Colombia	1200	98	8	6	15.3 (12.5; 18.6)	1.2 (0.6; 2.4)	0.9 (0.4; 2.0)	1.9 (1.1; 3.4)	0.1 (0; 1.1)
Mexico	750	44	8	1	17.8 (13.2; 23.9)	3.1 (1.6; 6.3)	0.4 (0.1; 2.8)	3.1 (1.6; 6.3)	0.4 (0.1; 2.8)
Puerto Rico	300	51	19	6	22.0 (16.7; 29.0)	7.7 (4.9; 12.0)	2.3 (1.0; 5.2)	8.1 (5.2; 12.5)	1.0 (0.8; 4.7)

* Defined as the number of new cases arising from defined population in specified time period divided by the total at risk person-time of observation.

† 20 participants experienced two episodes, i.e., there was a total of 255 episodes.

**Table 3:** Number of cases with an acute febrile episode with serologically probable and virologically confirmed dengue

		Virologically confirmed			
		Yes	No	Total	
Serologically probable					
	Yes	11	39	50	PPV*: 22%
	No	7	178	185	PVN†: 96.2%
Total		18	217	235	
Sensitivity: 61.1%					
Specificity: 82%					

* PPV = predictive positive value.

† PVN = predictive negative value.

**Table 4:** Number and percentages of seropositive subjects at baseline and at the end of the study*

Study countries/sites	Positive at baseline			Positive at the end of the study		
	n/*N*	%	95% CI	n/*N*	%	95% CI
All countries	1821/2588	70.4	(68.6; 72.1)	1839/2595	70.9	(69.1; 72.6)
Brazil	249/449	55.5	(50.7; 60.1)	263/452	58.2	(53.5; 62.8)
Colombia	1086/1174	92.5	(90.8; 93.9)	1079/1176	91.8	(90.0; 93.3)
Mexico	353/693	50.9	(47.1; 54.7)	352/695	50.6	(46.9; 54.4)
Puerto Rico	133/272	48.9	(42.8; 55.0)	145/272	53.3	(47.2; 59.4)

* n = number of subjects that met the specified criteria (IgG positive); *N* = number of subjects with a valid dengue IgG ELISA result; CI = confidence interval.
